# COP27 Climate Change Conference: Urgent Action Needed for Africa and the World

**DOI:** 10.5811/westjem.59258

**Published:** 2022-10-22

**Authors:** Gregory E. Erhabor, Aiah A. Gbakima, Abraham Haileamlak, Jean-Marie Kayembe Ntumba, James Kigera, Laurie Laybourn-Langton, Bob Mash, Joy Muhia, Fhumulani Mavis Mulaudzi, David Ofori-Adjei, Friday Okonofua, Arash Rashidian, Maha El-Adawy, Siaka Sidibé, Abdelmadjid Snouber, James Tumwine, Mohammad Sahar Yassien, Paul Yonga, Lilia Zakhama, Chris Zielinski

**Affiliations:** 1 West African Journal of Medicine; 2 Sierra Leone Journal of Biomedical Research; 3 Ethiopian Journal of Health Sciences; 4 Annales Africaines de Medecine; 5 Annals of African Surgery; 6 University of Exeter; 7 African Journal of Primary Health Care & Family Medicine; 8 London School of Medicine and Tropical Hygiene; 9 Curationis; 10 Ghana Medical Journal; 11 African Journal of Reproductive Health; Eastern Mediterranean Health Journal; Eastern Mediterranean Health Journal; Mali Médical; Journal de la Faculté de Médecine d’Oran; African Health Sciences; Evidence-Based Nursing Research; East African Medical Journal; La Tunisie Médicale; University of Winchester

## Wealthy nations must step up support for Africa and vulnerable countries in addressing past, present and future impacts of climate change

The 2022 report of the Intergovernmental Panel on Climate Change (IPCC) paints a dark picture of the future of life on earth, characterised by ecosystem collapse, species extinction, and climate hazards such as heatwaves and floods (1). These are all linked to physical and mental health problems, with direct and indirect consequences of increased morbidity and mortality. To avoid these catastrophic health effects across all regions of the globe, there is broad agreement—as 231 health journals argued together in 2021—that the rise in global temperature must be limited to less than 1.5^o^C compared with pre-industrial levels.

While the Paris Agreement of 2015 outlines a global action framework that incorporates providing climate finance to developing countries, this support has yet to materialise (2). COP27 is the fifth Conference of the Parties (COP) to be organised in Africa since its inception in 1995. Ahead of this meeting, we—as health journal editors from across the continent—call for urgent action to ensure it is the COP that finally delivers climate justice for Africa and vulnerable countries. This is essential not just for the health of those countries, but for the health of the whole world.

### Africa has suffered disproportionately although it has done little to cause the crisis

The climate crisis has had an impact on the environmental and social determinants of health across Africa, leading to devastating health effects (3). Impacts on health can result directly from environmental shocks and indirectly through socially mediated effects (4). Climate change-related risks in Africa include flooding, drought, heatwaves, reduced food production, and reduced labour productivity (5).

Droughts in sub-Saharan Africa have tripled between 1970–79 and 2010–2019 (6). In 2018, devastating cyclones impacted three million people in Malawi, Mozambique and Zimbabwe (6). In west and central Africa, severe flooding resulted in mortality and forced migration from loss of shelter, cultivated land, and livestock (7). Changes in vector ecology brought about by floods and damage to environmental hygiene has led to increases in diseases across sub-Saharan Africa, with rises in malaria, dengue fever, Lassa fever, Rift Valley fever, Lyme disease, Ebola virus, West Nile virus and other infections (8, 9). Rising sea levels reduce water quality, leading to water-borne diseases, including diarrhoeal diseases, a leading cause of mortality in Africa (8). Extreme weather damages water and food supply, increasing food insecurity and malnutrition, which causes 1.7 million deaths annually in Africa (10). According to the Food and Agriculture Organization of the United Nations, malnutrition has increased by almost 50% since 2012, owing to the central role agriculture plays in African economies (11). Environmental shocks and their knock-on effects also cause severe harm to mental health (12). In all, it is estimated that the climate crisis has destroyed a fifth of the gross domestic product (GDP) of the countries most vulnerable to climate shocks (13).

The damage to Africa should be of supreme concern to all nations. This is partly for moral reasons. It is highly unjust that the most impacted nations have contributed the least to global cumulative emissions, which are driving the climate crisis and its increasingly severe effects. North America and Europe have contributed 62% of carbon dioxide emissions since the Industrial Revolution, whereas Africa has contributed only 3% (14).

### The fight against the climate crisis needs all hands on deck

Yet it is not just for moral reasons that all nations should be concerned for Africa. The acute and chronic impacts of the climate crisis create problems like poverty, infectious disease, forced migration, and conflict that spread through globalised systems (6, 15). These knock-on impacts affect all nations. COVID-19 served as a wake-up call to these global dynamics and it is no coincidence that health professionals have been active in identifying and responding to the consequences of growing systemic risks to health. But the lessons of the COVID-19 pandemic should not be limited to pandemic risk (16, 17). Instead, it is imperative that the suffering of frontline nations, including those in Africa, be the core consideration at COP27: in an interconnected world, leaving countries to the mercy of environmental shocks creates instability that has severe consequences for all nations.

The primary focus of climate summits remains to rapidly reduce emissions so that global temperature rises are kept to below 1.5 °C. This will limit the harm. But, for Africa and other vulnerable regions, this harm is already severe. Achieving the promised target of providing $100bn of climate finance a year is now globally critical if we are to forestall the systemic risks of leaving societies in crisis. This can be done by ensuring these resources focus on increasing resilience to the existing and inevitable future impacts of the climate crisis, as well as on supporting vulnerable nations to reduce their greenhouse gas emissions: a parity of esteem between adaptation and mitigation. These resources should come through grants not loans, and be urgently scaled up before the current review period of 2025. They must put health system resilience at the forefront, as the compounding crises caused by the climate crisis often manifest in acute health problems. Financing adaptation will be more cost-effective than relying on disaster relief.

Some progress has been made on adaptation in Africa and around the world, including early warning systems and infrastructure to defend against extremes. But frontline nations are not compensated for impacts from a crisis they did not cause. This is not only unfair, but also drives the spiral of global destabilisation, as nations pour money into responding to disasters, but can no longer afford to pay for greater resilience or to reduce the root problem through emissions reductions. A financing facility for loss and damage must now be introduced, providing additional resources beyond those given for mitigation and adaptation. This must go beyond the failures of COP26 where the suggestion of such a facility was downgraded to “a dialogue” (18).

The climate crisis is a product of global inaction, and comes at great cost not only to disproportionately impacted African countries, but to the whole world. Africa is united with other frontline regions in urging wealthy nations to finally step up, if for no other reason than that the crises in Africa will sooner rather than later spread and engulf all corners of the globe, by which time it may be too late to effectively respond. If so far they have failed to be persuaded by moral arguments, then hopefully their self-interest will now prevail.
